# Nutrition education in type 2 diabetic patients: comparison of individual and collective care

**DOI:** 10.1186/1758-5996-7-S1-A179

**Published:** 2015-11-11

**Authors:** Marina Moreno Wardi, Maria Regina Calsolari, Pedro Weslley Souza do Rosário

**Affiliations:** 1 Santa Casa De Belo Horizonte, Belo Horizonte, Brazil

## Background

Individual care has helped health educators to recognize the needs of each patient, ensuring the achievement of established goals. Collective care has been related to the cost benefit and it has enabled positive psychosocial effects.

## Objective

To compare two groups with individual care (Group 1) or collective care (Group 2), by using the same approach techniques in nutrition to assess compliance with nutritional recommendations.

## Materials and methods

31 patients with type 2 diabetes were selected irrespective of gender, aged 40 to 75 yrs. and the mean duration of disease was 10 yrs. Patients were randomly separated into two groups, individual (group 1) or collective care (group 2). All patients attended a total of six meetings. First meeting was individual and consisted of signing Informed Consent Agreement, Biochemical tests (fasting glucose, glycated hemoglobin, total cholesterol and fractions, triglycerides), anthropometric data (weight, height, BMI and waist circumference) and questionnaire to asses dietary habits and knowledge about healthy diet for managing diabetes. During the second and fourth meetings patients was provided with information on diabetes and nutritional intakes. Subjects received a booklet with summary of the topics. The patients were asked to prepare questions based on the nutrition education for discussion. In the third and fifth meeting a discussion was performed using booklets and daily diet of each participant. The last meeting consisted of reevaluation of the data collected at the beginning. All the meetings with individual care followed the same characteristics of education in diabetes used with collective care. Wilcoxon test was used for statistical analysis.

## Results

**Figure 1:**
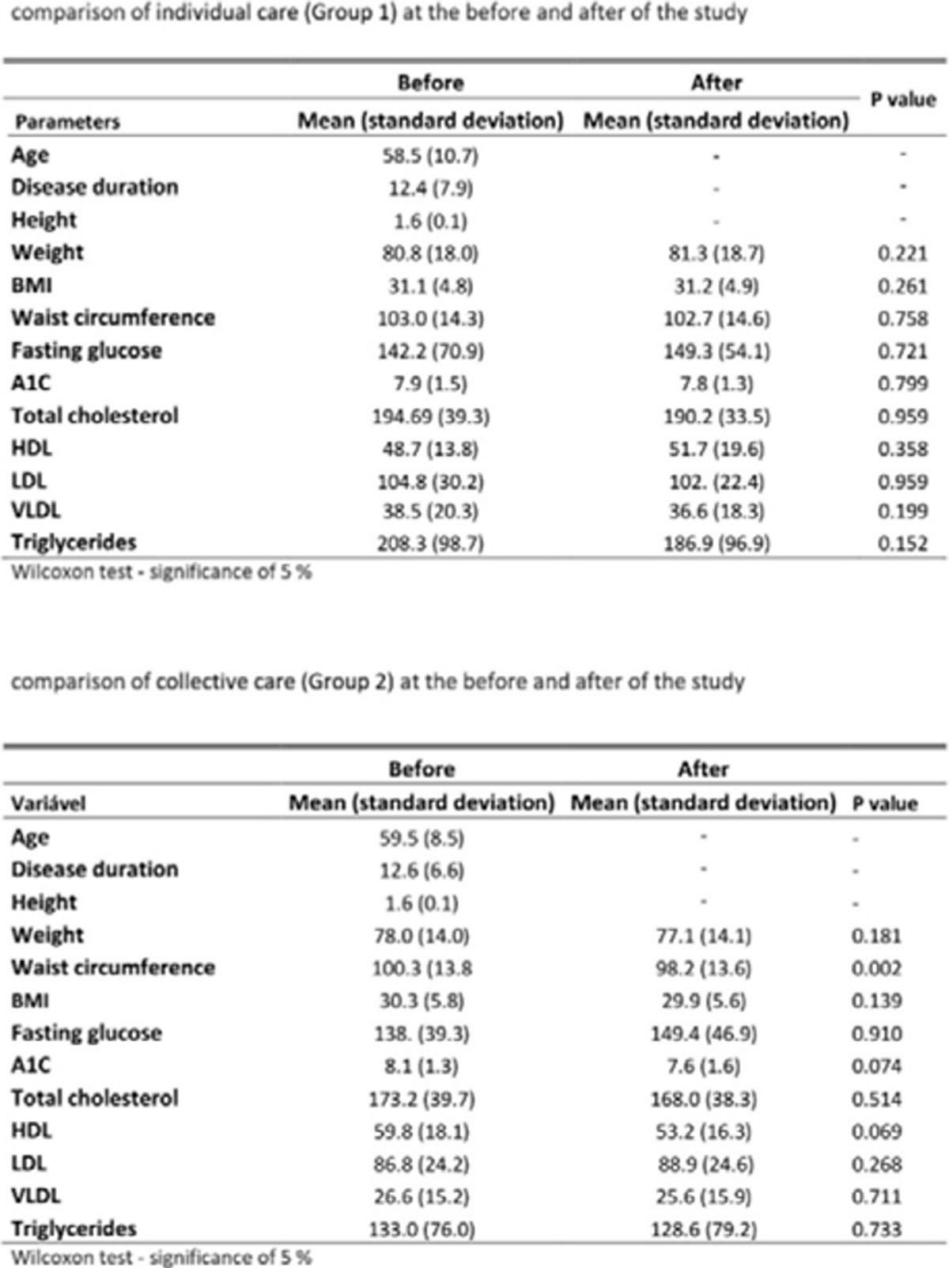
Comparison of individual care (Group 1) and collective care (Group 2) before and after the study.

**Figure 2:**
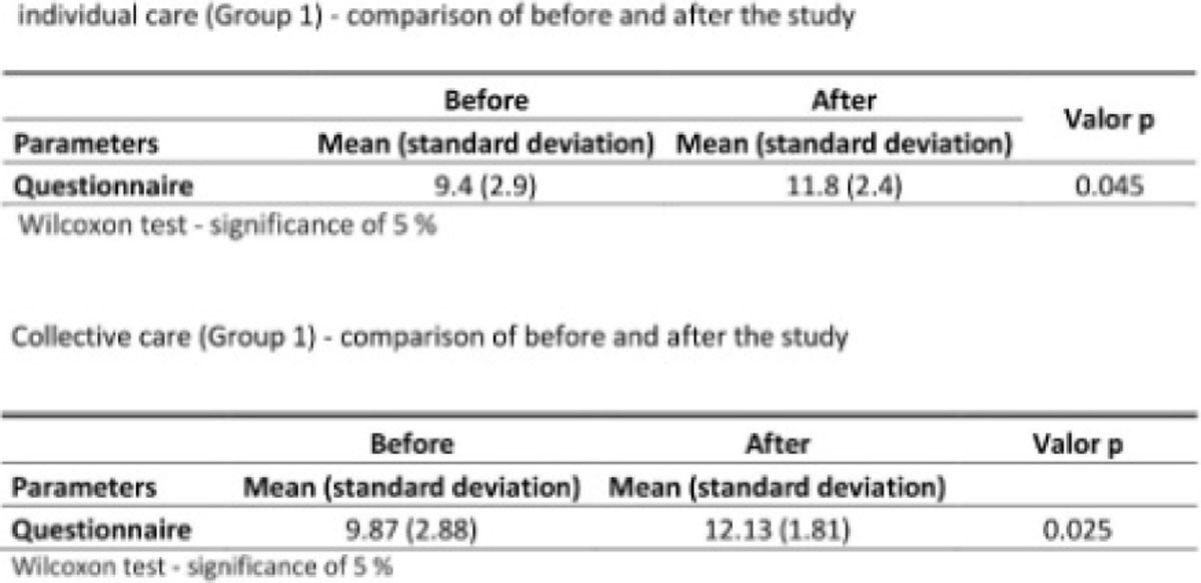
Questionnaire to assess dietary habits and knowledge about healthy diet for managing diabetes.

**Figure 3:**
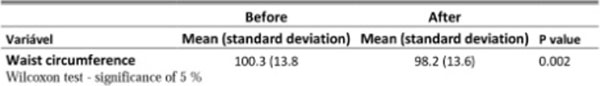
Reduction of waist circumference varied after the intervention in collective care (Group 2).

There was no significant difference between Group 1 and Group 2 regarding biochemical parameters, weight and BMI after the intervention (Figure 1). In the questionnaire of dietary habits, Group 1 and Group 2 have shown significant differences to perceptions of healthy eating (p=0.045 and p=0.025, respectively) (Figure 2). In Group 2 the reduction of waist circumference varied significantly (p=0.002) after the intervention (Figure 3).

## Conclusion

Nutrition education has been a positive impact on the treatment of diabetic patients and an important tool for health professionals.

